# Photochemically Altered Air Pollution Mixtures and Contractile Parameters in Isolated Murine Hearts before and after Ischemia

**DOI:** 10.1289/ehp.1306609

**Published:** 2013-10-22

**Authors:** Rachel McIntosh-Kastrinsky, David Diaz-Sanchez, Kenneth G. Sexton, Corey M. Jania, Jose Zavala, Stephen L. Tilley, Ilona Jaspers, M. Ian Gilmour, Robert B. Devlin, Wayne E. Cascio, Haiyan Tong

**Affiliations:** 1 Department of Environmental Sciences and Engineering, Gillings School of Global Public Health, University of North Carolina at Chapel Hill, Chapel Hill, North Carolina, USA; 2 Environmental Public Health Division, National Health and Environmental Effects Research Laboratory, U.S Environmental Protection Agency, Research Triangle Park, North Carolina, USA; 3 Department of Medicine, Division of Pulmonary and Critical Care Medicine; 4 Center for Environmental Medicine, Asthma, and Lung Biology, and; 5 Department of Medicine, Division of Cardiology, School of Medicine, University of North Carolina at Chapel Hill, Chapel Hill, North Carolina, USA

## Abstract

Background: The cardiopulmonary effects of the individual criteria air pollutants have been well investigated, but little is known about the cardiopulmonary effects of inhaled multipollutant mixtures that more realistically represent environmental exposures.

Objectives: We assessed the cardiopulmonary effects of exposure to photochemically altered particle-free multipollutant mixtures.

Methods: We exposed mice to filtered air (FA), multipollutant mixtures, or ozone (O_3_) for 4 hr in a photochemical reaction chamber. Eight hours after exposure, we assessed cardiac responses using a Langendorff preparation in a protocol consisting of 20 min of global ischemia followed by 2 hr of reperfusion. Cardiac function was assessed by measuring the index of left-ventricular developed pressure (LVDP) and contractility (dP/dt) before ischemia. On reperfusion after ischemia, recovery of postischemic LVDP and size of infarct were examined. We used bronchoalveolar lavage (BAL) cell counts to assess lung inflammation.

Results: Exposure to the multipollutant mixtures decreased LVDP, baseline rate of left ventricular contraction (dP/dt_maximum_), and baseline rate of left ventricular relaxation (dP/dt_minimum_) compared with exposure to FA. Exposure to O_3_ also decreased heart rate and dP/dt_minimum_. Time to ischemic contracture was prolonged in the multipollutant-mixture group relative to that in the FA group. Mice in the multipollutant-mixture group had better recovery of postischemic LVDP and smaller infarct size. Exposure to multipollutant mixtures and to O_3_ exposure increased numbers of macrophages in the BAL fluid.

Conclusions: Exposure to photochemically altered urban air pollution appears to affect cardiac mechanics in isolated perfused hearts. Inhalation of acute multipollutant mixtures decreases LVDP and cardiac contractility in isolated non-ischemic murine hearts, prolongs ischemic contracture, increases postischemic recovery of LVDP, and reduces infarct size.

Citation: McIntosh-Kastrinsky R, Diaz-Sanchez D, Sexton KG, Jania CM, Zavala J, Tilley SL, Jaspers I, Gilmour MI, Devlin RB, Cascio WE, Tong H. 2013. Photochemically altered air pollution mixtures and contractile parameters in isolated murine hearts before and after ischemia. Environ Health Perspect 121:1344–1348; http://dx.doi.org/10.1289/ehp.1306609

## Introduction

Epidemiological studies have linked acute and chronic ambient air pollution exposure with cardiovascular diseases and shown that air pollution exposure increases the risk of mortality, ischemic heart disease, heart failure, and arrhythmias (Brook et al. 2010). The Clean Air Act (1970) and the Clean Air Act Amendments (1990) established National Air Quality Standards for individual “criteria pollutants.” Consequently, air pollution health effects research studies have largely focused on characterizing the effects of exposure to these pollutants on an individual basis. However, “real-world” air pollution is far more complex than exposure to an individual agent because real-world air pollution contains freshly emitted primary aerosol as well as photochemically aged secondary aerosols formed in the atmosphere during the oxidation of gas-phase precursors (Kanakidou et al. 2005). There is a growing realization that a multipollutant experimental approach is needed to understand the relevant modes of action of ambient air pollutant mixtures on human health (Dominici et al. 2010). However, there is currently a paucity of data on how complex mixtures impact key target organ systems associated with morbidity and mortality. To fill this critical knowledge gap, we tested whether inhaled exposure to particle-free complex mixtures representative of gaseous mixtures found in urban environments can modulate pulmonary inflammation and cardiac mechanics.

We used an environmental photochemical reaction chamber located at the University of North Carolina at Chapel Hill to generate model multipollutant-mixture atmospheres. The chamber uses sunlight to imitate the natural photochemistry of urban mixtures and produce a combination of compounds that is similar in composition to that found in urban multipollutant mixtures (Jeffries 1995; Sexton et al. 2004). Previous *in vitro* studies have utilized these chambers to expose individual cell cultures to examine the toxicity of air pollution mixtures and have shown that exposure to photochemically altered particle-free urban mixtures causes significant inflammatory responses (Sexton et al. 2004) and greater genetic alterations (Rager et al. 2011) compared with exposure to primary urban mixtures.

Experimental studies have demonstrated the ability of individual air pollutants to cause cardiopulmonary toxicity in animals. For example, acute exposure to particulate matter can increase pulmonary and systemic inflammation and lung injury, cause vascular dysfunction, alter heart rate variability, induce arrhythmia, and enhance cardiac ischemic injury (Brook et al. 2010). We (Cho et al. 2009; Tong et al. 2010) and others (Cozzi et al. 2006) have shown that particulate matter exposure can enhance cardiac ischemia/reperfusion injury in animals. However, extrapolation to multipollutant mixtures is far from simple. For instance, the redox cycling potential of a mixture, which is thought to be an important predictor of generation of inflammation, may be very different than that of its constituent parts. To address this issue, we tested the hypothesis that photochemically aged particle-free multipollutant mixtures can cause inflammation and impair cardiac function. Using a murine model, we evaluated the effects of multipollutant-mixture exposure on cardiovascular and pulmonary end points. We report here that exposure to multipollutant mixtures at concentrations that produce only minimal pulmonary effects significantly affect cardiac function—including decreased left ventricular developed pressure and contractility—but unexpectedly reduce cardiac ischemia/reperfusion injury.

## Methods and Materials

*Experimental animals.* Mice were maintained at 22°C with a 12-hr light/dark cycle and free access to food (ProLab RMH 3000; PMI Nutrition, Saint Louis, MO) and water. We purchased 45 female C57BL/6 mice (5 months of age, mean weight 23.9 ± 0.39 g) from Jackson Laboratory (Bar Harbor, ME) and they were acclimated for 2 months before they were used. All experimental procedures were performed in compliance with protocols approved by the University of North Carolina at Chapel Hill Institutional Animal Care and Use Committee according to National Institutes of Health guidelines (National Research Council 2011). The animals were treated humanely and with regard for alleviation of suffering.

*Generation of photochemical urban mixtures.* The University of North Carolina at Chapel Hill’s outdoor environmental photochemical reaction chamber was used to generate exposure atmospheres. Synthetic Urban Mix (Scott Specialty Gases, Plumsteadville, PA), a volatile organic compound (VOC) mixture (Jeffries 1995), and NO_x_ [nitric oxide (NO) and nitrogen dioxide (NO_2_)] were used as the starting materials for the test atmosphere. The synthetic particle-free urban mixture contains 55 different hydrocarbons at specific ratios that represent chemicals present in urban atmospheres (Sexton et al. 2004). On the morning of the exposure, the chamber was humidified naturally by pre-flushing with HEPA-filtered ambient air. At 0700 hours, the Synthetic Urban Mix volatile organics were drawn from a gas cylinder into the photochemical reaction chamber while a liquid mixture containing less-volatile organics was injected into the chamber. NO_x_ was drawn from a gas cylinder (AirGas, National Welders, Morrisville, NC) into the chamber to establish a test atmosphere containing 2 ppm NO_x_.

Chemical constituents inside the chamber during the experiment were assessed by gas measurement methods as described previously (Rager et al. 2011). NO and NO_2_ levels were measured once per minute using a Teledyne model 9841 NO_x_ analyzer (Teledyne Monitor Labs, Englewood, CO). Ozone (O_3_) was measured every minute with a Teledyne model 9811 monitor. Concentrations of these compounds were averaged during the exposure. Other secondary products such as the carbonyl-containing aldehydes and ketones were measured every 15 min by gas chromatography and mass spectrometry. Because the O_3_ level was elevated to 0.243 ppm in the chamber, the same concentration of O_3_ was used in the single-pollutant O_3_ exposure. O_3_ was generated from oxidized air using an O_3_ generator (model OL80A; Ozone Services, Yanco Industries, Burton, British Columbia, Canada).

*Animal exposure.* Because the photochemical reaction depends on the weather condition and because the multipollutant mixtures generated are not reproducible, mice were exposed in groups to photochemically aged particle-free multipollutant mixtures (*n* = 15), 0.245 ppm O_3_ (*n* = 14), or filtered air (FA; *n* = 16) for 4 hr (2000–2400 hours) during their dark cycle on 3 separate days in an outdoor photochemical reaction chamber. One group of mice per exposure (*n* = 7 in the multipollutant-mixture group; *n* = 6 in the O_3_ group; *n* = 8 in the FA group) was used for the isolated heart perfusion and the other mice (*n* = 8 per group) were used for assessing lung inflammation.

*Cardiac function.* As described previously (Tong et al. 2009), 8–11 hr after exposure, mice were anesthetized with an intraperitoneal (ip) injection of sodium pentobarbital (80 mg/kg body weight). After injecting the mice with intravenous heparin (100 units), the hearts were excised rapidly and placed in ice-cold Krebs–Henseleit buffer. The aortas were cannulated and perfused retrograde at a constant pressure of 100 cmH_2_O. The non-recirculating perfusate was Krebs–Henseleit buffer containing 120 mmol/L sodium chloride, 5.9 mmol/L potassium chloride, 1.2 mmol/L magnesium sulfate, 1.75 mmol/L calclium chloride, 25 mmol/L sodium bicarbonate, and 11 mmol/L glucose. The buffer was aerated with 95% oxygen and 5% carbon dioxide, and maintained at pH 7.4 and 37°C.

For assessment of contractile function, a latex balloon on the tip of a polyethylene catheter was inserted through the left atrium into the left ventricle. The catheter was connected to a pressure transducer (model 041500503; Argon Medical Devices, Athens, TX) at the same height as the heart. The pressure of the left ventricular balloon was inflated to 0–5 cmH_2_O. We used a PowerLab data acquisition system (AD Instruments, Milford, MA) to collect and process the heart rate, left ventricular developed pressure [LVDP, i.e., LV peak minus end-diastolic pressure (LVEDP)], and contractility (dP/dt) data. All hearts had been perfused for 25 min when the baseline measurements were taken. We then initiated 20 min of global no-flow ischemia followed by 2 hr of reperfusion. Onset of ischemic contracture was detected when the left ventricular pressure began to increase during ischemia. We measured recovery of LVDP, expressed as a percentage of the initial pre-ischemic LVDP, at 40 min of reperfusion after 20 min of ischemia.

*Cardiac necrosis evaluation.* As described previously by Tong et al. (2009, 2010), at the end of 2 hr of reperfusion, we perfused the hearts with 15 mL of a 1% solution of 2,3,5-triphenyltetrazolium chloride (TTC) dissolved in Krebs–Henseleit buffer, incubated them in 1% TTC at 37°C for 10 min, and then fixed them in formalin. We measured the area of necrosis by taking cross-sectional slices through the ventricles, which were then photographed using a digital camera mounted on a stereo-microscope. We quantified the resulting images by measuring the areas of stained (viable tissue) versus unstained tissue (infarct) using Adobe Photoshop (Adobe Systems, San Jose, CA). Infarct size was expressed as a percentage of the total ventricular section and averaged from four images.

*Bronchoalveolar lavage.* Lung inflammation has been shown to be one of the pathways that mediate the cardiac effects (Brook et al. 2010); therefore, we examined whether exposure to multipollutant mixtures or to O_3_ resulted in lung inflammation. Twelve hours after exposure, mice were anesthetized with an ip injection of pentobarbital sodium (50 mg/kg). The lungs were lavaged five times with 1 mL 1× Hanks’ Balanced Salt Soultion (HBSS; Gibco, Life Technologies, Carlsbad, CA) and cellular components of the bronchoalveolar lavage (BAL) fluid were separated by centrifugation at 1,500 rpm for 10 min at 4^o^C. The cell pellet was resuspended in 500 μL HBSS and total cells were counted using a hemocytometer. Cytospin preparations were made and stained with Hema 3 (Fisher Scientific, Waltham, MA) to evaluate the BAL cellular composition.

*Statistical analysis.* Data are expressed as mean ± SE. Nonparametric analyses were performed because the data were not normally distributed. We performed comparisons among the multipollutant-mixture, O_3_, and FA control groups by Kruskal–Wallis *U*-test followed by Dunn’s multiple comparison test. We used the Mann–Whitney *U*-test to compare the multipollutant-mixture or O_3_ groups with the FA control group. The statistical significance level was set at *p* < 0.05.

## Results

*Composition of particle-free photochemically generated multipollutant mixtures.* Photochemical reactions of the original hydrocarbons and NO_x_ mixtures generated > 300 carbonyl secondary products. Among those products, the levels of detected VOC compounds estimated in the chamber are listed in Table 1. As reported by Rager et al. (2011), the average chamber levels of NO and NO_2_ decreased throughout the day, and levels of secondary chemical products such as O_3_, formaldehyde, and acetaldehyde increased. The photochemical chamber contained 0.243 ppm of O_3_ and secondary carbonyls. The formaldehyde level in the photochemical chamber was 5× higher than that in the O_3_ and FA control chambers, and the acetaldehyde level was elevated in the photochemical chamber but not detectable in the O_3_ and FA control chambers. No particulate matter or secondary organic aerosol was formed within the chamber.

**Table 1:** Average concentrations (ppb) of compounds measured in the photoreaction chamber.

Chemical	Pollutant exposure		
	Mixtures	O_3_	FA
O_3_	243	245	10
NO	0	0	1
NO_2_	18	4	7
CO	500	300	300
Peroxyacetyl nitrate	1.3	< 0.2	< 0.2
Formaldehyde	64.2	12.2	12.2
Acetaldehyde	22.1	0	0
Acetone	11.1	0	0
2-Hexanone	17.1	0	0
Glyoxal	11.7	0.6	0.6
Methylglyoxal	15.7	0.8	0.8
Unknown cluster	74.9	9.5	9.5
Estimated total carbonyls	286.0	23.1	23.1
Total organic precursors	2,000	< 20	< 20
Alkanes (23 total)	1,133
Isopentane	173
*n*-Butane	147
Propane	92
Ethane	77
Alkenes (16 total)	262
Ethene	53
2,3,3-Trimethyl-1-butene	32
*cis*-2-Pentene	27
*trans*-2-Butene	23
Aromatics (13 total)	605
Toluene	138
1,2,4-Trimethylbenezene	112
*m*-Xylene	74
Benzene	44
CO, carbon monoxide; FA, filtered air; mixtures, multi­pollutant mixtures. O_3_, NO, NO_2_, and CO were measured every minute ­during the exposure period and averaged for reporting. The other compounds were measured every 15 min during the exposure period. All samples were measured by gas chromatography and mass spectrometry, using a direct cryogenically trapped sample taken from the chamber. Because the exposures were conducted in the dark, concentrations were stable and homogeneous.			

**Figure 1:**
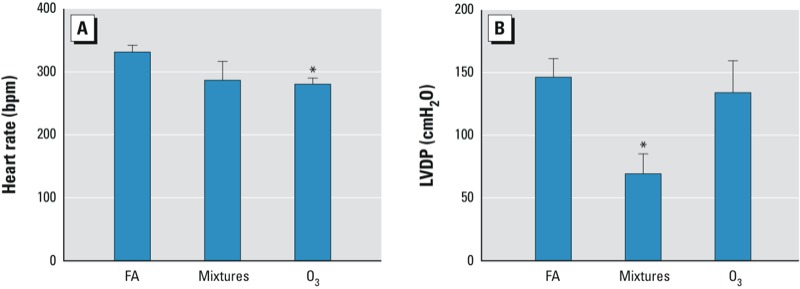
Heart rate and cardiac function in isolated perfused murine hearts before ischemia. Heart rate (*A*) and LVDP (*B*) at baseline prior to ischemia in murine hearts isolated 8–11 hr after inhalation exposure to filtered air (FA), multi­pollutant mixtures (mixtures), or O_3_ for 4 hr as described in “Materials and Methods.” (*n* = 8 in the FA group, *n* = 6 in the O_3_ group, and *n* = 7 in the mixtures group.) ^*^p < 0.05, compared with FA control group by Kruskal–Wallis U-test followed by Dunn’s multiple comparison.

**Figure 2:**
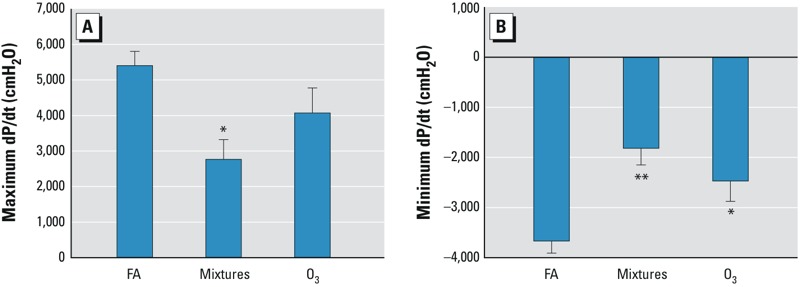
Multi­pollutant-mixture and O_3_ inhalation reduced cardiac contractility in isolated, perfused murine hearts. Cardiac contractility assessed by maximum (*A*) and minimum (*B*) dP/dt at baseline prior to ischemia in murine hearts isolated 8 hr after inhalation exposure to FA (*n* = 8), mixtures (*n* = 7), or O_3_ (*n* = 6) for 4 hr as described in “Materials and Methods.“ ^*^p < 0.05, and ^**^p < 0.01, compared with FA control group by Kruskal–Wallis U-test followed by Dunn’s multiple comparison.

*Cardiac effects.* When compared with the FA control, the baseline heart rate prior to ischemia decreased with O_3_ exposure, whereas heart rate was unchanged in the hearts of mice exposed to the particle-free multipollutant mixtures (Figure 1A). We observed no differences in baseline coronary artery flow rate at constant pressure between the multipollutant-mixture or O_3_ groups compared with the FA control group (Table 2). However, hearts from the multipollutant-mixture group had lower baseline LVDP (69.2 ± 16.0 cmH_2_O) compared with the FA group (146.4 ± 14.8 cmH_2_O; *p* < 0.05) although there was no significant difference between the FA- and O_3_-groups (134.2 ± 5.4 cmH_2_O) (Figure 1B). Exposure to multipollutant mixtures or O_3_ decreased baseline left ventricular contractility. The baseline rate of contraction (dP/dt_maximum_; maximum first derivative of the change in left ventricular pressure/time) was lower in the multipollutant-mixture group (2,764 ± 558 cmH_2_O/sec) compared with the FA group (5,405 ± 400 cmH_2_O/sec; *p* < 0.05) (Figure 2A). Yet, the change in dP/dt_maximum_ did not differ between the O_3_ (4,070 ± 704 cmH_2_O/sec) and FA group. The baseline rate of relaxation (dP/dt_minimum_; minimum first derivative of the change in left ventricular pressure/time) was decreased by exposure to the multipollutant mixtures (–1,822 ± 335 cmH_2_O/sec; *p* < 0.01) and O_3_ exposure (–2,477 ± 407 cmH_2_O/sec; *p* < 0.05) when compared with the FA control (–3,675 ± 242 cmH_2_O/sec) (Figure 2B).

**Table 2:** Hemodynamic measures (means ± SEs) of isolated mouse hearts.

Experimental group	FA (*n *= 8)	O_3_ (*n *= 6)	Mixtures (*n *= 7)
Baseline condition
Heart rate (bpm)	331 ± 10	280 ± 10*	286 ± 30
LVDP (cmH_2_O)	146.4 ± 14.8	134.2 ± 25.4	69.2 ± 16.0*
dP/dt_maximum_ (cmH_2_O/sec)	5,405 ± 400	4,070 ± 704	2,764 ± 558*
dP/dt_minimum_ (cmH_2_O/sec)	–3,675 ± 242	–2,477 ± 407*	–1,822 ± 335**
Coronary flow rate (mL/min)	2.0 ± 0.2	1.6 ± 0.3	1.8 ± 0.2
At 40 min of reperfusion
Heart rate (bpm)	290 ± 21	278 ± 28	280 ± 23
LVDP (cmH_2_O)	33.5 ± 11.4	86.5 ± 34.9	38.0 ± 8.8
dP/dt_maximum_ (cmH_2_O/sec)	1,418 ± 467	2,963 ± 1212	1,612 ± 352
dP/dt_minimum_ (cmH_2_O/sec)	–801 ± 257	–1,751 ± 674	–872 ± 150
Coronary flow rate (mL/min)	1.6 ± 0.1	1.2 ± 0.2	1.5 ± 0.2
dP/dt_maximum_, maximum first derivative of the change in left ventricular pressure/time; dP/dt_minimum_, minimum first ­derivative of the change in left ventricular pressure/time. *p < 0.05, and **p < 0.01, compared with FA control group by Kruskal–Wallis *U*-test followed by Dunn’s multiple ­comparison.			

**Figure 3:**
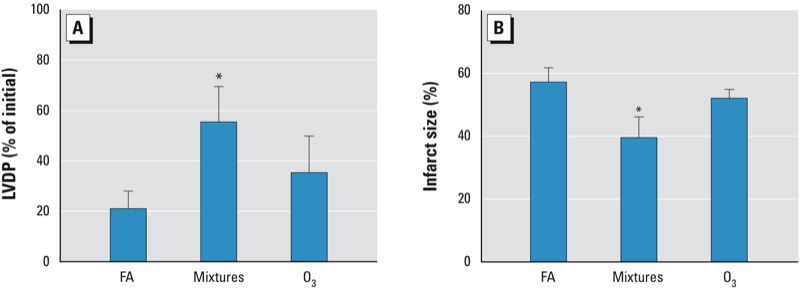
Multi­pollutant-mixture exposure improved recovery of post­ischemic cardiac function and reduced size of infarct in murine hearts. (*A*) Recovery of LVDP, expressed as a percentage of the initial baseline pre-ischemic LVDP, was measured after 20 min of ischemia and 40 min of reperfusion in mouse hearts isolated 8 hr after inhalation exposure to FA (*n* = 8), mixtures (*n* = 7), or O_3_ (*n* = 6) for 4 hr as described in “Materials and Methods.“ (*B*) Infarct size, expressed as a percentage of the total ventricular section, was measured after 20 min of ischemia and 2 hr of reperfusion as described in “Materials and Methods.“ ^*^p < 0.05, compared with FA control group by Kruskal–Wallis U-test followed by Dunn’s multiple comparison.

Compared with the FA group (13.2 ± 1.4 min), time to ischemic contracture was not affected in the O_3_ group (14.0 ± 1.8 min), but was prolonged in the multipollutant-mixture group during the 20 min of ischemia (17.3 ± 0.5 min; *p* < 0.05). There was also an increase in postischemic recovery of LVDP at 40 min after reperfusion in the multipollutant-mixture group compared with the FA control group (70.7 ± 23.7% for multipollutant mixtures vs. 21.0 ± 7.1% for FA; *p* = 0.05) (Figure 3A). Infarct size was smaller in the multipollutant-mixture group (39.6 ± 6.6%) compared with the FA control (57.3 ± 4.6% for FA; *p* < 0.05) (Figure 3B). By 40 min of reperfusion, heart rate, LVDP, contractility, and coronary flow rate were not different among the multipollutant-mixture, O_3_, and FA groups (Table 2).

**Figure 4:**
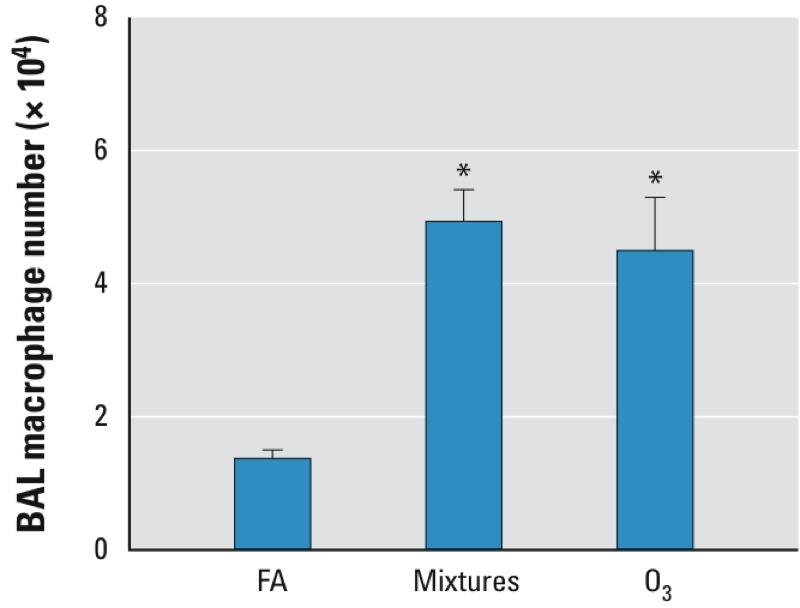
Multi­pollutant-mixture and O3 exposure increased macrophage number in BAL fluid. Macrophage number in BAL 12 hr after exposure to FA, multi­pollutant mixtures (mixtures), or O_3_ for 4 hr as described in “Materials and Methods“ (*n* = 8 per group). ^*^p < 0.05, compared with FA control group by Kruskal–Wallis U-test followed by Dunn’s multiple comparison.

*Airway inflammation.* Compared with the FA controls, the BAL from mice exposed to the multipollutant mixtures or O_3_ showed an increase in the number of macrophages (Figure 4). However, we found no influx of neutrophils or other cell types after any exposure.

## Discussion

Although our understanding of the health effects of single pollutants has advanced considerably over the last several decades, knowledge of the effects of multipollutant mixtures is limited. In the present study we used a photochemical reaction chamber to generate particle-free urban-like multipollutant mixtures for the purpose of evaluating the pulmonary and cardiac responses in mice to inhalation of an atmosphere containing complex multipollutant mixtures or O_3_ at the same concentration as present in the multipollutant mixtures. Thus, any difference in measured responses between these two exposures can be ascribed to effects of the multipollutant mixtures. Short-term inhalation of photochemically altered particle-free multipollutant mixtures and O_3_ alone depressed cardiac contractility (dP/dt) in the isolated perfused heart. However, exposure to the particle-free multipollutant mixtures delayed the onset of ischemic contracture and preserved contractile function during reperfusion in isolated mouse hearts. In addition, although we found no significant difference in the mechanical responses between the multipollutant mixtures and O_3_ alone, the magnitude of the effect of O_3_ alone was always less than the effect of the multipollutant mixtures. Thus, it appears that one or more of the multipollutant-mixture’s component(s) contribute to an additional effect beyond that of O_3_ alone.

Experimental studies by Gordon (2007) implicated particulate mass and the physicochemical properties of air pollutants as determinants of the health effects of air pollution inhalation. In particular, organic components of air pollution are thought to play an important role in affecting biological responses (Castranova et al. 2001). In the present study, mice were exposed to particle-free photochemically altered products of hydrocarbons and NO_x_, indicating that the cardiac effects measured following exposure resulted from gaseous components of multipollutant mixtures. We have previously exposed cultured lung cells to similar multipollutant mixtures in the photochemical reaction chamber and showed that gaseous products elicited biological and biochemical responses in the absence of particles (Sexton et al. 2004). Furthermore, animal studies have shown that spontaneously hypertensive and mildly cardiomyopathic rats exposed to filtered diesel exhaust exhibited either a similar degree or a greater magnitude of electrophysiological changes compared with exposure to whole diesel exhaust (Carll et al. 2012; Lamb et al. 2012), implying that gaseous components of air pollution might be driving the cardiovascular effects.

As shown previously (Sexton et al. 2004), levels of secondary chemical products such as O_3_, formaldehyde, acetaldehyde, and many volatile and semivolatile organic hydrocarbons increase as the photochemical reaction progresses. Therefore, the animals in our study were exposed to higher levels of secondary products, which may elicit biological effects on the cardiovascular system. Secondary products formed during the photochemical reactions can induce more robust inflammatory responses (Sexton et al. 2004) and greater genomic changes (Rager et al. 2011) in cultured lung cells. Secondary products, such as O_3_ generated during the photochemical reactions, may have contributed to the biological effects, including the O_3_-induced heart rate and baseline rate of left ventricular relaxation changes that we report here. Epidemiological studies have linked short-term O_3_ exposure with cardiovascular and respiratory mortality in 95 large U.S. communities (Bell et al. 2004). O_3_ exposure has also been associated with increased hospital admissions for heart failure (Yang 2008). Most recently, a controlled human-exposure study (Devlin et al. 2012) demonstrated that short-term O_3_ exposure increased pulmonary and systemic inflammation, altered autonomic control of heart rhythm, and induced changes in blood proteins involved in fibrinolysis. In addition, an animal study (Chuang et al. 2009) demonstrated that 5 days (8 hr/day) of 0.5 ppm O_3_ exposure in mice altered heart rates and mean blood pressure, inhibited endothelial-dependent vasorelaxation, and induced mitochondrial damage and atherogenesis.

The levels of the secondary products, formaldehyde and acetaldehyde, increased during the photochemical process in this study. Formaldehyde and acetaldehyde occur naturally in the environment and are produced in forest fires, automobile exhaust, and tobacco smoke. The ambient formaldehyde concentration was in the range of 0.4–7.5 ppb with a mean value of 2.2 ppb in New York City in the summertime in 2009 (Lin et al. 2012) and the ambient concentration of acetaldehyde averaged 5 μg/m^3^ (National Toxicology Program 2011). In the present study, exposure to formaldehyde may have contributed to the observed cardiac-depressive activity of the inhaled particle-free multipollutant mixtures. Similarly, acetaldehyde, another component of the multipollutant mixtures, could also affect cardiac function. Acetaldehyde has been shown to disrupt calcium ion (Ca^2+^) handling in the myocardium and to disturb cardiac excitation-contraction coupling (Oba et al. 2008), leading to reduced cardiac contractility. Therefore, the cardiac-depressive effects of inhalation of the multipollutant mixtures used in this study may have resulted from acetaldehyde-induced myocardial toxicity. Above all, the respiratory responses to volatile and semivolatile organic components of multipollutant mixtures, such as O_3_, have been investigated in the past; yet the cardiovascular effects of those components are not well known. More research is needed to evaluate the cardiovascular effects of exposure to these ubiquitous air contaminants.

The mechanistic basis for the cardiac-mechanical effects of exposure to multipollutant mixtures and O_3_ was not explored in this study. However, there are several pathways by which the air pollution mixtures could affect the cardiovascular system (Brook et al. 2010). Oxidative stress from multipollutant-mixture components such as O_3_ could cause systemic oxidative stress, resulting in myocardial contractile dysfunction. In addition, activation of pulmonary receptors could initiate a neurocardiogenic effect, producing an intracardiac response affecting cardiac cellular function. It is also possible that some components of multipollutant mixtures might translocate into the circulation, with attendant direct oxidative effects on the heart and vasculature. We detected only macrophage accumulation in BAL in this study, suggesting that airway inflammation may not mediate the cardiac effects of inhalation to multipollutant mixtures or to O_3_.

The decreased LVDP and cardiac contractility, delayed ischemic contracture, and preserved contractility during reperfusion consequent to exposure to inhaled multipollutant mixtures could indicate altered intracellular Ca^2+^ regulation in the myocardium. The pro-redox components of multipollutant mixtures could modulate the cardiac myocytes’ Ca^2+^ handling by reducing the intracellular Ca^2+^, or cause a change in the sensitivity of the contractile proteins to Ca^2+^, resulting in decreased cardiac contractility. On the other hand, the reduced intracellular Ca^2+^ overload during ischemia is associated with the preservation of mitochondrial function and adenosine triphosphate stores (Kowalchuk and Nesto 1989), as suggested by the multipollutant mixtures’ induced delay of ischemic contracture and better recovery of postischemic LVDP and smaller infarct size during reperfusion. Future studies are needed to better understand the role of intracellular Ca^2+^ regulation in mediating cardiovascular physiological effects from air pollution exposure.

In this study we used a perfused isolated heart model to evaluate the ventricular function. However, isolated heart models lack innervation and blood supply and, therefore, lack influence by autonomic and hormonal control: This could alter findings in intact hearts.

## Conclusion

Inhalation of particle-free photochemically altered multipollutant mixtures affects cardiac function in isolated mouse hearts. Specifically, inhalation of multipollutant mixtures depressed cardiac contractility in isolated non-ischemic hearts, and delayed ischemic contracture and preserved cardiac contractility in reperfused hearts, while eliciting mild pulmonary inflammation evidenced only by macrophage accumulation. Future studies are needed to identify the active components of photochemical reaction products responsible for the effects on myocardial mechanical performance as well as the mechanisms that underlie the cardiac effects.
